# Women’s midlife: the front line of Alzheimer prevention

**DOI:** 10.1172/JCI199832

**Published:** 2026-03-16

**Authors:** Lisa Mosconi

**Affiliations:** 1 Departments of Neuroscience, Neurology and Radiology, Weill Cornell Medicine, New York New York, USA.; 2 Wellcome Leap, San Diego California, USA.

## Abstract

Nearly two-thirds of patients with Alzheimer disease (AD) are women, most of them postmenopausal. While sex differences in AD have historically been attributed to women’s relative longevity, accumulating evidence challenges that view, pointing to female sex–specific biological underpinnings. In particular, neuroendocrine aging and the hormonal shifts that accompany the menopause transition have emerged as potentially modifiable AD risk factors in women. Yet, key neuroendocrine aging-related factors linked to increased AD and dementia risk, such as early menopause, premenopausal bilateral oophorectomy, frequent vasomotor symptoms, and midlife cognitive and mood disturbances, remain underinvestigated. Additionally, although a growing evidence base highlights the potential of menopause hormone therapy for AD prevention, particularly in women undergoing oophorectomy, progress remains hindered by a lack of clinical trials and biomarker-driven studies. This Review calls for a paradigm shift: from viewing AD risk as a byproduct of generalized aging to validating midlife neuroendocrine aging as a distinct window of vulnerability, and an opportunity for prevention. By 2050, over 1.2 billion women worldwide will be in or approaching menopause. The stakes are global, and the opportunity is urgent: to redefine AD prevention through sex-specific, time-sensitive, and biologically informed strategies that translate science into scalable, actionable care.

## Women and Alzheimer risk

Alzheimer disease (AD) is the most common cause of dementia, affecting over 55 million individuals worldwide (1), with projections exceeding 150 million by 2050 (2). Women (throughout the text, the term “women” refers to cisgender women) bear a disproportionate share of this burden, comprising nearly two-thirds of all AD cases, with the majority being postmenopausal women (3).

Since the 1990s, we have known that, after advanced age, female sex is the strongest risk factor for late-onset AD (4, 5). The estimated lifetime risk for a 45-year-old woman is 1 in 5 — twice that of a man of the same age (3). While risk increases for both sexes at age 65, the relative difference persists (5). AD is also the number 1 cause of death (COD) for women over 65, the only leading COD that kills more women than men (6), and one of the only two age-related neurodegenerative diseases with a higher female-to-male sex ratio, alongside multiple sclerosis (7). Disparities in AD are therefore not fully explained by women’s longer life expectancy or conventional risk profiles (8–12). Whether they reflect underlying biological differences or diagnostic bias, however, remains under debate.

From an epidemiological perspective, “risk” encompasses both prevalence and incidence. As higher prevalence in the absence of incidence differences may reflect longer survival after diagnosis, incidence data are central to the discussion. While some studies report higher incidence of AD and dementia (13–21), as well as progression from mild cognitive impairment (MCI) to AD (22–24) in women, others do not (25–30). Differences in study design, sample size, population age structure, follow-up duration, and diagnostic criteria may account for this variability. For instance, studies that included older participants (aged 75+) and longer follow-up periods, such as the Kungsholmen Project in Sweden and the PAQUID cohort in France (14, 16), found statistically significant higher AD incidence in women. In contrast, studies with younger baselines, shorter follow-ups, and primarily US-based populations, like the Framingham Study and Chicago Health and Aging Project (25, 27), were more likely to report no differences. Meta-analyses of pooled estimates support increased AD and dementia incidence in women (15, 31–33), with global age- and education-adjusted hazard ratios (HRs) indicating a modest but consistent excess risk (HR 1.12, 95% CI 1.02–1.23) (33). Disparities appear more pronounced in low- and middle-income countries (HR 1.73, 95% CI 1.25–2.39) (33), suggesting contributions from both biological and socioenvironmental factors, such as income level, educational attainment, health care access, and chronic disease burden.

Still, much of the prevalence-versus-incidence debate reflects a view of AD as a disease of old age, diagnosed late in its course, whereas AD is now understood as a disease of midlife, with symptoms that emerge years to decades later (34). Although the average age at diagnosis is 72 (35), the underlying pathology — amyloid-β (Aβ) deposition, neurofibrillary tangles, and neurodegeneration — may begin as early as between ages 45 and 65 (34). This discovery has transformed the field, moving from diagnosis-based approaches to biology-based frameworks emphasizing preclinical detection (34, 36). With the advent of biomarkers — brain imaging, cerebrospinal fluid (CSF), and blood-based biomarkers (BBBs) — it is now possible to detect hallmark AD pathology years before symptoms appear. This has pinpointed midlife not only as a critical window for biological disease onset but also for sex divergence in risk trajectories. Viewed through this lens, the longstanding focus on late-life incidence may overlook the earlier, female sex–specific processes that set the stage for AD risk in women.

## Midlife neuroendocrine aging in AD risk

Neuroendocrine aging, and particularly the midlife transition to menopause, have emerged as biologically plausible and increasingly studied pathways linking female sex to AD risk. Menopause is a neuroendocrine transition that impacts multiple organ systems, including the brain (37). The transition involves a coordinated hormonal cascade marked by declining ovarian estradiol production, rising pituitary gonadotropins — luteinizing hormone (LH) and follicle stimulating hormone (FSH) — and changes in hypothalamic-pituitary-gonadal regulation (38). The average age of menopause, typically around age 51–52, aligns with the onset of the preclinical phase of AD (37) and the rise in lifetime risk in women (3). Decades of preclinical studies indicate that declines in sex steroid hormones, especially 17β-estradiol, contribute to the initiation of AD pathology in cellular and animal models (9, 11, 37, 39–43). Estradiol promotes neuronal resilience by reducing inflammation, suppressing tau phosphorylation, and enhancing non-amyloidogenic amyloid precursor protein (APP) processing, in turn reducing Aβ-induced neurotoxicity (37, 43–46). Conversely, estradiol deprivation accelerates neurodegeneration in female animals (47–50), while concurrent elevations in FSH and LH also promote amyloidogenic processes (51–54).

Clinically, several lines of evidence link early menopause, especially due to bilateral oophorectomy, with a higher risk of AD and all-cause dementia (40, 55–57). Whether later-onset, spontaneous menopause constitutes an AD risk factor remains debated. Several cross-sectional biomarker studies have been conducted in midlife women stratified by menopause status, e.g., premenopausal (regular menstrual cycles), perimenopausal (irregular menstrual cycles), and postmenopausal (no cycles for >12 consecutive months). Postmenopausal, and to a lesser extent perimenopausal, women exhibited increased AD biomarker burden compared with premenopausal women and age-controlled men, including higher brain Aβ (58–61) and tau levels (62), lower cerebral glucose metabolism, and lower gray matter volume (GMV) and white matter volume (58–61, 63–65) in AD-vulnerable regions. Additional findings include postmenopausal changes in brain mitochondrial ATP production (60, 66) and higher white matter hyperintensity (WMH) burden (67). These differences remained statistically significant adjusting for age, suggesting endocrine changes rather than age alone underlie the disparities. However, since postmenopausal women are typically older than premenopausal controls, group differences may still reflect aging rather than menopause per se. While longitudinal studies are scarce, two reported higher rates of Aβ accumulation, hypometabolism, and hippocampal volume loss in postmenopausal women compared with premenopausal women and age-controlled men (59, 60).

Sex differences have also been noted in AD biomarker progression in older age, with more severe outcomes for postmenopausal women than men of similar age (8–11). Female patients with AD exhibit greater rates of global and hippocampal atrophy (68–71) and neurofibrillary tangle burden than male AD patients with similar Aβ levels (72, 73). Interestingly, women exhibit higher verbal memory scores than men even after an AD diagnosis (74–76), possibly masking the earlier pathogenesis.

Taken together, these findings suggest an earlier onset of AD pathology in women, proximate to the menopause transition. Based on preclinical findings, this is generally attributed to the loss of ovarian sex steroid hormones. However, causality remains to be established, as most mechanistic evidence stems from animal studies and translational data are correlational in nature. Large-scale longitudinal studies are needed to map biomarker trajectories across the transition and into clinical disease, and determine whether neuroendocrine processes initiate AD pathology, accelerate its progression, or unmask preexisting vulnerabilities.

## Menopause hormone therapy and AD risk

Some of the debate around menopause’s role in AD risk stems from studies of menopause hormone therapy (MHT). While it is biologically plausible that restoring estrogen levels through MHT could help mitigate AD onset, clinical findings have been inconsistent (see *MHT* below). This has created uncertainty, in part due to the misconception that a risk factor is only valid if an effective intervention exists. In fact, risk factors are defined by mechanistic plausibility and biological relevance, not by the availability of a proven therapy (77).

For example, menopause is a recognized risk factor for cardiovascular disease (CVD) (78, 79), even though MHT is not approved for CVD prevention. This designation is based on increased CVD incidence following menopause (78, 79), and mechanistic evidence linking estrogen withdrawal to adverse effects on vascular tone, lipid metabolism, and inflammation (78, 79). Parallels between CVD and AD include the following: (a) AD risk is similar in men and women before age 45, then doubles in women (3); (b) early menopause is linked to greater AD and dementia risk (40, 55–57); and (c) estradiol loss affects synaptic function, Aβ/tau regulation, and brain bioenergetics; gonadotropins may promote amyloidogenic processes (Table 1).

When it comes to MHT effects, the Women’s Health Initiative (WHI) — the only randomized controlled trial (RCT) powered for hard CVD outcomes (heart attack, stroke) — found no overall benefit and possible harm when treatment was initiated more than 10 years after menopause (80). Trials in younger women, such as the Kronos Early Estrogen Prevention Study (KEEPS) and Early vs. Late Intervention Trial with Estradiol (ELITE), found MHT improved surrogate CVD markers like atherosclerosis progression and carotid intima-media thickness (81–83), highlighting the importance of timing. However, secondary outcomes are not regarded as conclusive for regulatory approval of preventive indications.

Similar considerations apply to AD. In the WHI-Memory Study (WHIMS), late-life MHT initiation had neutral or harmful effects on dementia incidence (84, 85), again mirroring CVD findings. While RCTs testing midlife MHT use are lacking, observational studies (86–88) and RCT biomarker data (89) indicate associations between midlife MHT use and reduced AD and dementia risk, especially among hysterectomized women (see *MHT* below).

## Identifying those at risk

**Figure 1:**
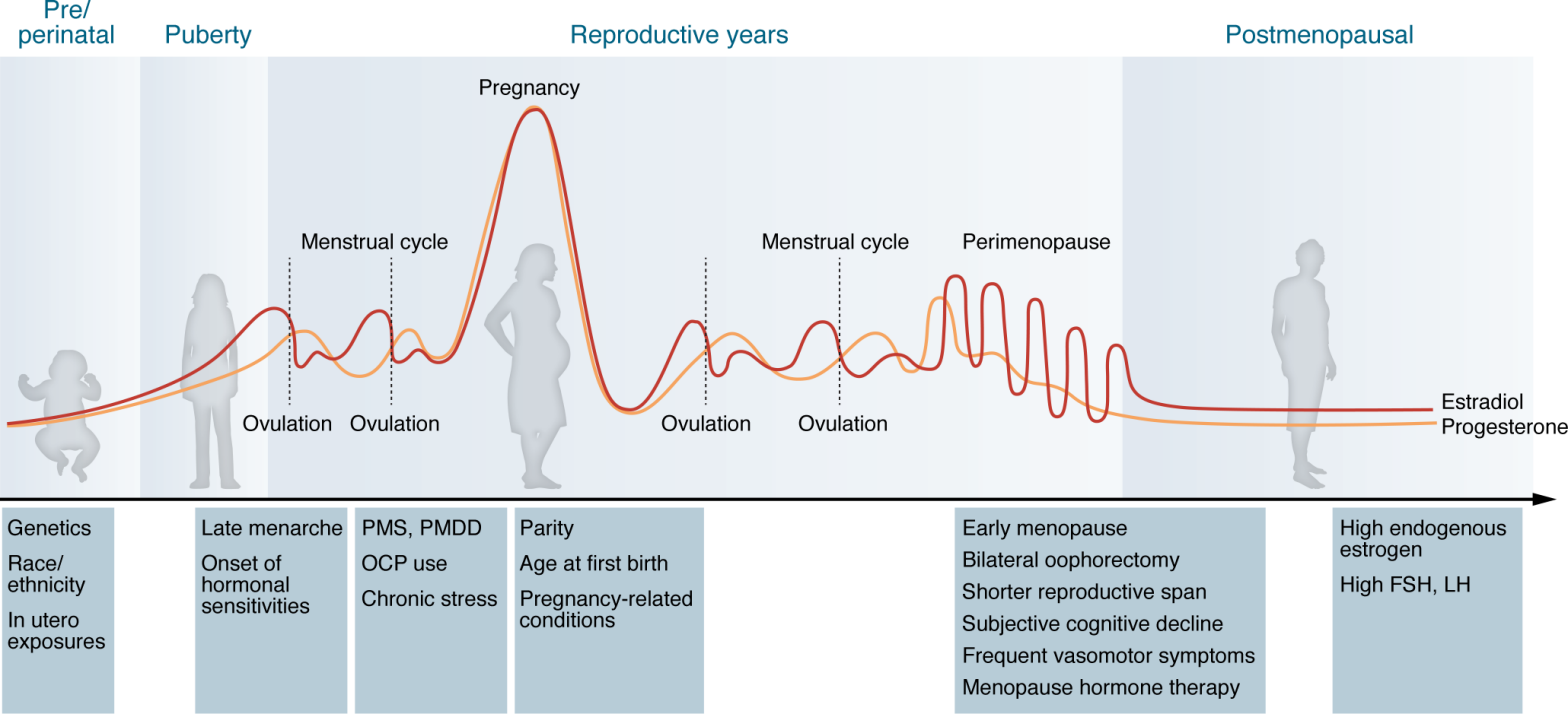
Neuroendocrine and reproductive health factors associated with AD risk across women’s lifespans. Neuroendocrine and reproductive health factors associated with AD risk across women’s lifespans. Estrogen exposure, reproductive milestones, and hormone sensitivity across the lifespan influence long-term brain health and AD risk in women. Key windows, such as puberty, pregnancy, and menopause, coincide with major hormonal transitions that can influence cognition, mood, and neuroplasticity. Genetic, lifestyle, and environmental factors further interact with reproductive history to shape brain aging trajectories. FSH, follicular stimulating hormone; LH, luteinizing hormone; OCP, oral contraceptives; PMS, premenstrual syndrome; PMDD, premenstrual dysphoric disorder.

Converging biological, epidemiological, and clinical evidence positions neuroendocrine aging and menopause as contributors to female AD risk, underscoring the need to identify vulnerable subgroups and inform effective interventions. As reviewed below, several female sex–specific neuroendocrine and reproductive health factors have been linked to increased risk for AD and all-cause dementia (Figure 1).

## Early menopause

For most women, menopause occurs as a result of spontaneous follicular depletion and subsequent ovarian insufficiency, which are associated with declines in estradiol and progesterone and increases in FSH and LH. Menopause can also occur at earlier ages due to premature ovarian insufficiency (POI) or be induced by bilateral oophorectomy (BO), chemotherapy, or radiation therapy (90).

An earlier onset of menopause has been consistently linked with a higher risk of AD and all-cause dementia (42, 91). A meta-analysis of 22 studies involving nearly 5 million women found that later menopause (after age 55) was associated with 33% reduced risk of AD, 13% reduced risk of all-cause dementia, and 18% reduced risk of cognitive impairment (42). Another meta-analysis confirmed these findings, showing a 22% higher risk of dementia or cognitive decline with early menopause, and a 7% reduced risk with late menopause (92). A third meta-analysis found increased dementia risk with both early menopause (37%) and POI (18%) (93).

Multiple studies have reported nearly double the long-term risk of dementia following BO (40, 55–57), which leads to abrupt cessation of sex hormone production, and smaller but statistically significant increases after unilateral oophorectomy or hysterectomy without oophorectomy (56, 94). While BO at any age is associated with an 8% increased risk of dementia (92), risk increases substantially with younger age at the time of BO, especially before age 45 (56, 95), with some studies reporting up to 70% higher risk (96).

Induced iatrogenic menopause by means of hormone-modulating therapy (HMT) (e.g., endocrine or antiestrogen therapy used to treat breast cancer) has also been examined, as these therapies block or suppress estrogen action (97). Evidence directly linking HMT to AD risk is limited and mixed, with studies reporting positive, negative, and null associations (98). However, recent large-scale analyses indicate that treatment with selective estrogen receptor modulators (SERMs) (e.g., tamoxifen) and steroidal aromatase inhibitors (AIs) (e.g., exemestane) is associated with reduced incidence of AD and neurodegenerative disease (99). A Medicare-based study similarly reported lower AD and dementia incidence among women treated with SERMs and AIs, but not among those treated with selective estrogen receptor degraders (SERDs), when treatment began before age 75 (100).

Although less studied in relation to AD, SERMs exhibit tissue-specific effects by differentially binding to estrogen receptor (ER) subtypes (ERα and ERβ) and coregulators (101). In breast tissue, they block ERα to inhibit proliferation, while in the brain they primarily act on ERβ, reducing Aβ accumulation and inflammation (101). Among AIs, steroidal agents are associated with lower AD risk, whereas nonsteroidal AIs (e.g., anastrozole, letrozole) showed the opposite (99). This may reflect the mild androgenic activity of steroidal AIs, which may promote neuroprotection by enhancing androgen signaling and local brain estrogen synthesis — effects absent in nonsteroidal AIs (101). These findings merit further investigation.

## Late menarche

Age at menarche has drawn interest as a potential factor in AD risk, with the hypothesis that earlier menarche may confer neuroprotection via longer lifetime estrogen exposure. Since age at menarche is not consistently associated with age at menopause, it is examined as an independent factor. The largest study to date, involving 4 million women, found that later menarche was indeed associated with increased the risk of dementia (102). However, other studies have reported generally neutral associations (10, 103–110). Meta-analytic findings also diverge; one study reported a 15% increased risk of dementia or cognitive decline in those with late menarche (≥16 vs. <13 years) (92), while another found no categorical association using a 14-year cutoff, but observed a statistically significant J-shape curve in dose-response meta-analysis, with increased risk at the later extreme of menarche timing (42). Differences in the age thresholds used to define early or late menarche likely contribute to these discrepancies.

## Shorter reproductive span

A woman’s reproductive lifespan (the interval between menarche and menopause) has also been examined in relation to dementia risk, under the premise that a longer reproductive span reflects greater cumulative estrogen exposure. Again, findings are mixed. One meta-analysis found that shorter reproductive span (≤34 years) was associated with a 14% increased dementia risk (relative risk [RR] 1.14, 95% CI 1.05–1.24), while longer span (≥38 years) was linked to a 9% reduced risk (RR 0.91, 95% CI 0.83–0.99) (92). Another meta-analysis reported neutral effects in categorical comparisons, but identified an inverse relationship between reproductive duration (≥35 vs. <35 years) and dementia in dose-response analyses (42). These discrepancies may reflect methodological differences in defining reproductive duration or population-level differences in age at menarche and menopause.

## Polycystic ovary syndrome and endometriosis

There is limited research on common reproductive endocrine disorders such as endometriosis (a chronic estrogen-dependent condition characterized by the growth of endometrium-like tissue outside the uterus) and polycystic ovary syndrome (PCOS, a hormonal disorder marked by hyperandrogenism and ovulatory dysfunction) in relation to AD risk. In a Canadian population–based cohort study, women with PCOS developed dementia nearly two decades earlier than controls (111). In contrast, current evidence does not support a direct association between endometriosis and AD risk (112).

## Other reproductive factors

Parity — the number of times a woman has given birth — generally shows a nonlinear relationship with AD risk (10, 11). Several studies report an inverted U-shape association, where having 1–4 children is linked with lower dementia risk (106, 113, 114), while grand multiparity (≥5 children) is associated with increased risk (106, 115, 116). A meta-analysis found that both nulliparous women (RR 1.11, 95% CI 1.06–1.16) and those with 5 or more children (RR 1.28, 95% CI 1.15–1.44) had increased dementia risk, with each additional childbirth increasing risk by 3% (92). Some studies also suggest associations between longer cumulative time spent pregnant or breastfeeding and reduced AD risk (103, 114, 117), although others report the opposite (102). Since estrogen levels are suppressed during lactation, these effects may involve different mechanisms.

Age at first childbirth has received less attention, but a cohort study of more than 4 million women found that older first-time mothers had a lower risk of dementia than those who gave birth in their 20s (118). The underlying mechanisms are unclear and may reflect differences in socioeconomic status, stress exposure, and lifestyle rather than biological factors alone (118). Maternal age at childbirth has also been linked to AD risk in offspring, with both young (15–19 years) and older (40+) maternal ages associated with increased AD risk in their children (119, 120).

Finally, pregnancy complications, including preeclampsia, gestational diabetes, preterm births, and peri/postpartum depression, remain understudied in relation to AD risk.

## Low estrogen, elevated gonadotropins

The increased risk of AD associated with menopause is often attributed to the loss of estradiol’s neuroprotective effects. However, meta-analyses of observational studies show that higher total estradiol levels after menopause are associated with increased risk of dementia (RR 1.46, 95% CI 1.15–1.85) (92). These findings suggest that after prolonged estrogen deprivation, the brain may become less responsive or resistant to estrogen’s effects.

In parallel, preclinical research implicates pituitary gonadotropins in AD pathogenesis (51, 52). Unlike estradiol, which fluctuates before stabilizing at low levels after menopause, FSH and LH levels increase steadily across the menopause transition, with over 10-fold and 3-fold increases, respectively (121). Mechanistic studies suggest that early rises in gonadotropins may act as upstream regulators of AD pathology, supporting the “timing hypothesis” that positions menopause as a critical inflection point for neurodegenerative risk (51, 52). Translational and observational human data remain limited, but emerging findings link higher FSH and LH to increased risk of MCI and dementia (122, 123) and higher AD biomarker load in midlife women (124, 125).

## Subjective cognitive decline

Subjective cognitive decline (SCD), which is defined as self-reported decline in memory, concentration, and mental acuity not meeting criteria for clinical impairment, is reported by 34% to 62% of peri- and postmenopausal women (38, 126). This is noteworthy, since memory decline is the earliest neuropsychological predictor of AD (127, 128), and individuals reporting SCD, especially women (129, 130), are at higher risk of progression to dementia and exhibit AD biomarker abnormalities already in midlife (131–134).

While the mechanisms linking SCD to menopause and AD remain under investigation, clinical studies consistently show that women in the perimenopausal and early postmenopausal stages exhibit subtle but measurable declines in verbal memory, attention, and processing speed (135–140). These effects are independent of vasomotor symptoms (VMS), disturbed sleep, and mood symptoms (141, 142). Imaging studies further support a biological basis; SCD in postmenopausal women has been associated with lower medial temporal lobe volume, disrupted functional connectivity (130, 143), and altered mitochondrial ATP production in AD-vulnerable brain regions (144). Together, these findings support the view that menopause-related SCD may serve as an early clinical marker of AD risk.

## Frequency of VMS

Emerging research indicates associations between menopausal VMS (hot flashes) and AD biomarker risk. In a study of 226 midlife women, more frequent objectively monitored VMS during sleep, but not in daytime, were associated with greater white matter hyperintensity volume (WMHV), a marker of possible cerebrovascular injury (145). A subsequent study from the same cohort found that sleep-related VMS were associated with lower plasma Aβ_42/40_ ratios, suggesting higher brain Aβ burden (146). While these cross-sectional findings suggest VMS may serve as a marker of underlying neurovascular vulnerability, further research is needed to determine whether they reflect or contribute to mechanisms relevant to AD.

## Genetics

The apolipoprotein E (APOE) ε4 allele is the strongest genetic risk factor for late-onset AD, conferring a higher risk in women than in men (4, 5, 147). Estrogen modulates *APOE* expression (148), potentially impacting MHT outcomes. Meta-analyses of observational studies provide contrasting findings of reduced AD or dementia incidence with MHT among *APOE-4* carriers (149) or noncarriers (88). In the KEEPS trial, among *APOE-4* carriers, transdermal estrogen (tE2) therapy was associated with lower Aβ load compared with both placebo and oral conjugated equine estrogens (CEEs) (89). No effects were observed among noncarriers (89). Observational research also points to reduced tau burden and more favorable Aβ dynamics in MHT users, particularly among *APOE-4* carriers (150, 151).

ER polymorphisms are also of interest, as estrogen exerts its effects via ERα (ESR1), ERβ (ESR2), and the G protein–coupled estrogen receptor (GPER1). A 10-year longitudinal study found that genetic variations in all three ER variants were associated with cognitive decline and increased tau pathology, with *GPER1* variants showing the most consistent effects (152). Additionally, *ESR2* and *ESR1* polymorphisms, such as the PvuII variant in *ESR1*, have been linked to a higher risk of AD or dementia (153, 154). These findings underscore the need for genotype-informed analyses of MHT for AD risk reduction.

## Race and ethnicity

Hispanic and Black women are at a higher risk for dementia (3) and also tend to experience more severe and frequent VMS compared with White women (155, 156). These findings point to disparities in both menopausal symptoms and dementia risk. However, further research is needed to determine whether these differences reflect underlying biological vulnerability or other factors.

## Hormone therapies

### MHT.

Growing evidence linking menopause to AD risk in women has renewed interest in the potential preventive role of MHT. This has been a subject of debate for decades, mainly due to the absence of definitive “Level 1” RCT evidence demonstrating risk-reduction effects.

To date, the only RCT to directly assess dementia outcomes is the WHIMS trial (84, 85). WHIMS enrolled postmenopausal women aged 65–79 (mean age 71 years), free from dementia at baseline. Participants were randomized to either estrogen-only therapy (ET: 0.625 mg/day of oral CEEs for women with hysterectomy) or estrogen-progestogen therapy (EPT: 0.625 mg/day CEEs + 2.5 mg/day of oral medroxyprogesterone acetate [MPA] for women with an intact uterus) versus placebo (84, 85). With fewer AD cases than expected, the trial shifted its primary outcome to all-cause dementia. Results showed a 2-fold increase in dementia incidence in the EPT group (HR 2.05, 95% CI 1.21–3.48) (84), and a nonsignificant 49% risk increase in the ET group (HR 1.49, 95% CI 0.83–2.66) (85). The incidence of MCI did not differ significantly between treatment and placebo groups (EPT HR 1.07, 95% CI 0.74–1.55; ET HR 1.34, 95% CI 0.95–1.89) (84, 85). A major critique of WHIMS is its focus on older women without menopausal symptoms, likely missing the therapeutic window for estrogen efficacy (157). The findings are also not generalizable to current prescribing practices, which emphasize symptom relief closer to menopause and often use different formulations, including tE2 and micronized progesterone. Typical use of MHT would rarely involve starting therapy at ages above 65. Additionally, as only 47% (ET) and 50% (EPT) of dementia cases in WHIMS were classified as AD, with the rest being vascular or mixed dementia (84, 85), results are also not directly generalizable to AD.

Given the approximately 20-year gap between menopause and AD symptom onset, RCTs testing whether midlife MHT reduces late-life AD incidence are unlikely to be feasible. In their absence, real-world data and observational studies of early postmenopausal women provide insights. Recent meta-analyses report that MHT, mainly ET, initiated near menopause is associated with an 11%–30% reduced risk of AD and all-cause dementia (87, 88, 149). In contrast, consistent with WHIMS findings, initiation of therapy more than 10 years after menopause is linked to neutral or increased risk, depending on formulation (87, 88, 149). Together, RCTs and observational data indicate increased risk with MHT started in old age, and a possible benefit when initiated early. However, observational findings require cautious interpretation due to inherent biases. Confounding by “healthy user bias” is a central concern; women prescribed MHT tend to be healthier, more highly educated, and socioeconomically advantaged than non-users (158). Thus, MHT may serve as a proxy for better health care access or healthier lifestyle, rather than being causally beneficial. Although most studies adjust for education and socioeconomic status, residual confounding is possible. “Confounding by indication” is another concern, as MHT users may experience more severe menopausal symptoms, such as VMS, which themselves have been independently linked to increased dementia risk (37).

Additional insights come from RCTs assessing cognitive performance, particularly verbal memory, which is among the earliest neuropsychological indicators of AD (137). Meta-analysis of 34 RCTs found that MHT, primarily ET for women with hysterectomy, improved global cognition compared with placebo (86). When initiated near menopause, ET improved verbal memory, while late-life initiation had no effects (86). For EPT, midlife use was neutral, while late-life use was associated with improved verbal memory but reduced global cognition (86). Cognitive changes, however, are not specific to AD, and most neuropsychological tests are designed to detect impairment in older adults, not the subtler changes that may occur in midlife. This increases the risk of ceiling effects and may hinder detection of improvement (86). Notably, in the KEEPS-Cog trial, while no cognitive benefits or harm were observed, early postmenopausal women randomized to tE2 exhibited lower brain Aβ plaque load compared with placebo (89).

Finally, concerns have been raised that, even if protective, MHT’s benefit may not justify widespread use given high number-needed-to-treat (NNT) estimates. These are based on an 18-year follow-up of the WHI, which reported reduced AD-related mortality in CEE users and neutral effects in CEE/MPA users (159), yielding NNTs of approximately 2,000 for CEEs and approximately 2,500 for CEE+MPA to prevent one AD-related death. However, these figures pertain to mortality, not AD risk reduction. To date, no NNT estimates exist for MHT in reducing AD risk, whether for CEE/MPA or other regimens.

It is increasingly clear that continued reliance on data derived from WHI-era formulations and populations is limiting. As the field advances, there is a need to refocus scientific and clinical efforts on generating an evidence base that reflects current prescribing practices, considers individual risk profiles, and leverages biological markers to directly assess the impact of MHT on AD risk. Mixed results from RCTs and observational studies of AD incidence point not to the failure of the concept, but to a failure of precision, highlighting the need for clinical and biomarker-driven stratification to determine which women might benefit, when, and how. In this light, MHT needs to be reevaluated not as a one-size-fits-all preventive tool, but rather as a targeted intervention whose safety and efficacy depend on timing, individual risk profile, and underlying biology.

The “timing hypothesis” posits that MHT may offer neuroprotective effects if initiated early, generally within 10 years of menopause or before age 60, but may be neutral or harmful if started later. Reflecting these considerations, while current guidelines do not recommend MHT for the prevention of cognitive decline or dementia in the general population, they do support initiating estrogen therapy near the time of menopause to help preserve cognitive function in women with early menopause, especially due to oophorectomy (160). The value of MHT for other women remains debated. A key limitation to testing the window-of-opportunity hypothesis is the absence of validated biomarkers to directly assess MHT’s effects on brain estrogen activity. Estrogen concentrations in plasma and brain are not strongly correlated (161), and to date, no study has quantified how MHT alters brain estrogen levels or their downstream effects on AD pathology. Recent advances in PET imaging with selective ER ligands represent a promising step forward (162), enabling measurements of brain ER expression in vivo and real-time monitoring of estrogenic treatments in future clinical trials.

### Other hormonal therapies.

Oral contraceptives (OCPs) suppress ovulation and lower circulating levels of FSH and LH. Beyond their contraceptive role, OCPs can be used to alleviate menopausal symptoms, although evidence of long-term neurological effects remains limited. Some MRI studies have reported greater GMV in OCP users compared with never-users (163), although findings have been inconsistent (10).

Gonadotropin-releasing hormone (GnRH) agonists, used clinically for endometriosis and some premenopausal hormone-sensitive breast cancer, suppress FSH secretion. In animal studies, they were shown to reduce Aβ pathology and improve cognitive performance (53). A clinical trial of leuprolide acetate (Lupron) in 109 women with mild to moderate AD reported cognitive benefits among those already receiving an acetylcholinesterase inhibitor (AChEI), suggesting a synergistic effect (164). These preliminary findings reinforce the need for further investigation into hormonal interventions as potential modifiers of AD risk.

## Validating neuroendocrine and reproductive risk factors in women

Advancements in AD biomarkers and analytical tools, alongside deeper insights into estrogen’s action in brain, are reshaping how we identify women at risk. Biomarker abnormalities — chiefly Aβ and tau measured via positron emission tomography (PET) imaging, CSF, and BBBs — are now part of the defining criteria for the preclinical AD stage (34, 36). Besides being widely used in research, amyloid-PET is approved for the differential diagnosis of AD and serves as a primary endpoint in RCTs of anti-amyloid therapies (165). Tau PET is also gaining traction, and plasma Aβ_42/40_ and phosphorylated tau isoforms (p-tau181, p-tau217) hold promise for scalable, noninvasive screening (166), although their prognostic value is under investigation (166). In the context of this Review, AD biomarkers provide a window into midlife, enabling earlier detection, risk stratification, and monitoring of disease progression and therapeutic response in women undergoing endocrine shifts. Other biomarkers of interest include indicators of synaptic activity, mitochondrial function, neurovascular integrity, and neuroinflammation.

Multiple lines of evidence support the menopause transition as a system-level biological inflection point for AD risk, and therefore an opportunity to extend neuroprotection for those at risk. Within this framework, estrogen withdrawal emerges as a modifiable risk factor for AD, potentially offset by estrogen therapy when delivered as a time-sensitive, biologically informed intervention, and particularly when guided by genetic, endocrine, and biomarker profiles. These considerations align with a broader shift in the field, from care to prevention, emphasizing modifiable risks that can be targeted early to alter AD trajectory.

**Figure 2:**
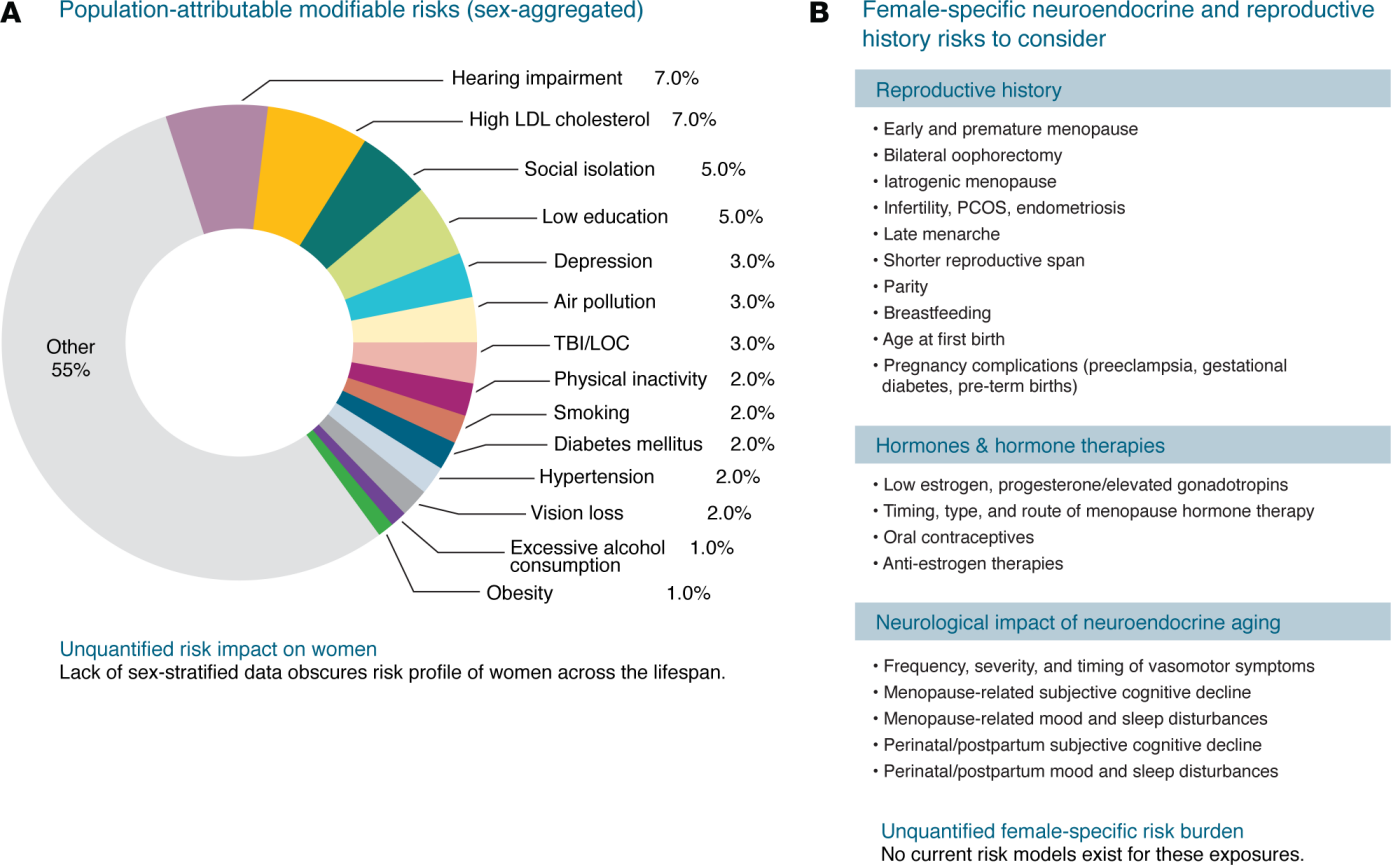
AD modifiable risk factors: current population models omit female sex–specific risk burden. (**A**) Current AD population-attributable risk (PAR) models estimate that 40%–45% of cases are due to modifiable risk factors (based on data from ref. 77), but these estimates are based on sex-aggregated data and fail to account for any female sex–specific neuroendocrine exposures. TBI/LOC, traumatic brain injury/loss of consciousness. (**B**) In contrast, a growing list of plausible female sex–specific neuroendocrine and reproductive history risks may help explain the sex disparity in AD and inform precision prevention strategies for women.

While factors like age, sex, and genetics cannot be changed, several modifiable factors — from cardiovascular and metabolic health to physical and cognitive activity — are estimated to account for up to 45% of global AD cases (77, 167) (Figure 2). However, current population-attributable risk (PAR) models are sex-aggregated, and female sex–specific risks remain unquantified, despite growing evidence that individual risk factors differ in strength and prevalence between the sexes (33, 168). Furthermore, no current PAR estimates exist for female sex–specific neuroendocrine and reproductive history factors (Figure 2), many of which are modifiable through hormone-based or other emerging therapies. As a result, prevention frameworks remain one-size-fits-all, underestimating women’s cumulative risk burden, and overlooking actionable opportunities for sex-specific intervention.

Closing this gap requires robust, sex-specific biomarkers, deeper understanding of how female sex influences AD biology, and stage-specific interventions tailored to women’s aging trajectories. To achieve this, future research must move beyond association and provide hard evidence of causal pathways linking neuroendocrine changes to AD risk in women, chiefly through deep phenotyping of female sex–specific risk factors paired with biomarker-defined outcomes. This will require the application of causal inference methodologies such as directed acyclic graphs (DAGs), structural equation modeling, and Mendelian randomization. Large-scale time-to-event (TTE) analyses using survival models such as Cox proportional hazards or flexible parametric models can further quantify the risk of progressing to AD biomarker positivity as a function of endocrine transitions, reproductive timing, hormone exposure, genotype, and more. These approaches require harmonized, multimodal data across observational cohorts, large-scale biorepositories, national registries, and electronic health records (EHRs). Ovarian hormone suppression studies, such as those using GnRH agonists or antagonists, offer a naturalistic model for investigating the cognitive and biomarker consequences of induced, yet reversible, hypoestrogenism. Simultaneously, the field should prioritize interventional studies that test whether hormonal therapies, including different formulations and routes, and novel nonhormonal therapies such as neurokinin 3 receptor (NK3R) antagonists, can reduce AD biomarker burden when initiated in midlife. Given the long preclinical phase of AD, shorter-term RCTs using AD biomarkers as primary endpoints offer a feasible and accelerated path to evidence. Complementary quasi-experimental designs using real-world data (e.g., target trial emulation, propensity-score matching, inverse probability of treatment weighting) can strengthen the evidence base in the absence of long-term RCTs. Together, these strategies would enable the field to isolate modifiable neuroendocrine mechanisms and develop precision prevention strategies for women at risk for AD.

By 2050, over 1.2 billion women worldwide will be in or near menopause. Intervening at midlife — when neuroendocrine transitions begin and prevention potential is highest — offers an opportunity to redefine AD risk management. Recognizing menopause as a neurologically active process reframes AD prevention as a sex-specific challenge requiring early detection and personalized strategies. Tailored, biomarker-driven strategies targeting female sex–specific biology could shift the trajectory of AD at scale, reducing suffering and global burden.

## Funding support

This work is the result of NIH funding, in whole or in part, and is subject to the NIH Public Access Policy. Through acceptance of this federal funding, the NIH has been given a right to make the work publicly available in PubMed Central.

- Wellcome Leap CARE (Cutting Alzheimer’s Risk through Endocrinology).
- NIH/National Institute on Aging grant P01AG026572.
- Maria Shriver’s Women’s Alzheimer’s Movement.
- Philanthropic support to the Weill Cornell Medicine Alzheimer’s Prevention Program.

## Figures and Tables

**Table 1:**
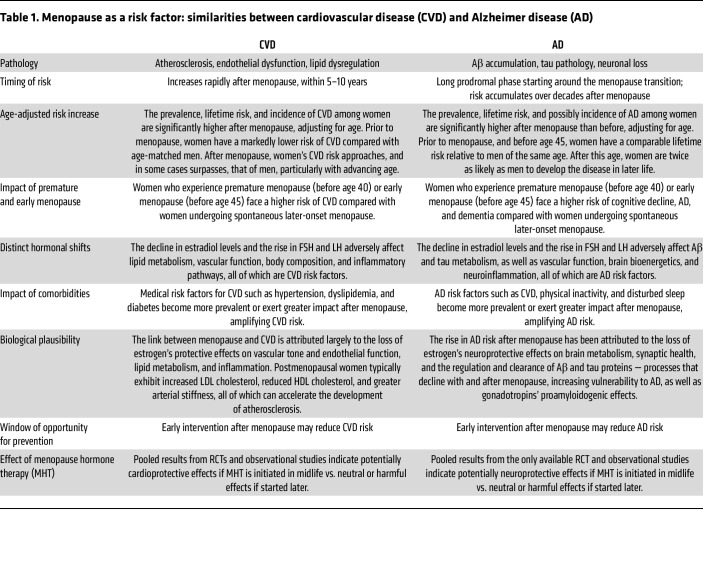
Menopause as a risk factor: similarities between cardiovascular disease (CVD) and Alzheimer disease (AD)

## Version 1 03/16/2026

Electronic publication
